# Metastatic Bellini Duct Carcinoma: A Case Report and Literature Review

**DOI:** 10.7759/cureus.98287

**Published:** 2025-12-02

**Authors:** Houda El Maoudda, Othmane Zouiten, Amina Mohtaram, Leila Afani, Mohamed El Fadli, Rhizlane Belbaraka

**Affiliations:** 1 Department of Medical Oncology, Centre Hospitalo-Universitaire Mohammed VI de Marrakech, Marrakesh, MAR

**Keywords:** bellini collecting tubules, cancer, chemotherapy, metastases, poor prognosis

## Abstract

Collecting duct carcinoma (CDC) of Bellini is a very rare subtype of renal cell carcinoma, generally associated with poor prognosis due to delayed diagnosis and limited sensitivity to conventional therapies used in renal cell carcinoma. We report the case of a 53-year-old female patient who presented with progressive lower back pain and chronic cough. A contrast-enhanced CT urogram revealed a large mass in the left kidney. The patient underwent radical nephrectomy. Histopathological examination confirmed a diagnosis of CDC of the kidney, with positive staining for cytokeratin 7 and epithelial membrane antigen. Staging investigations revealed pulmonary metastatic disease. The patient is receiving first-line chemotherapy with gemcitabine and cisplatin and responded favorably to treatment, showing clinical and radiological stability after three cycles.

## Introduction

Bellini duct carcinoma, also known as collecting duct carcinoma (CDC) of the kidney, is a rare and aggressive malignant tumor that accounts for less than 1% [1] of all renal cancers, highlighting the rarity of this histological subtype. Arising from the collecting ducts of the renal excretory system, this carcinoma is characterized by its medullary origin, infiltrative behavior, and generally poor prognosis due to delayed diagnosis and limited sensitivity to conventional therapies used in renal cell carcinoma. We report a clinical case of a patient diagnosed with metastatic Bellini duct carcinoma.

## Case presentation

We report the case of a 53-year-old female patient with no significant medical history. The onset of symptoms dates back four months. The clinical history was notable for a chronic dry cough and intermittent lower back pain, without hematuria or other associated signs. These symptoms developed in the context of a worsening general condition. On initial clinical examination, tenderness was noted on palpation over the left lumbar region, with tension in the left flank. The remainder of the physical examination was unremarkable.

**Figure 1:**
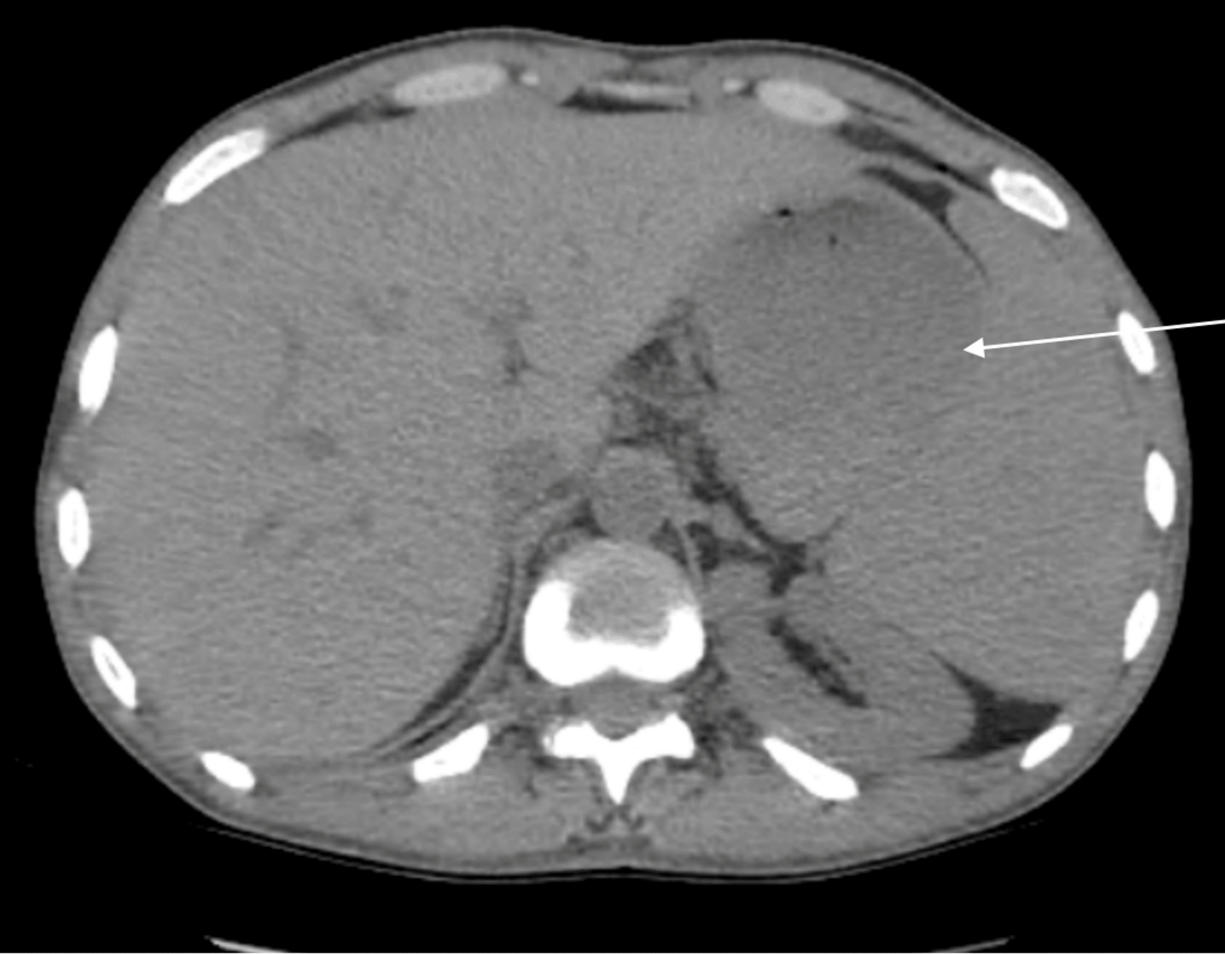
Axial CT urogram image demonstrating a large left mid-renal mass

**Figure 2:**
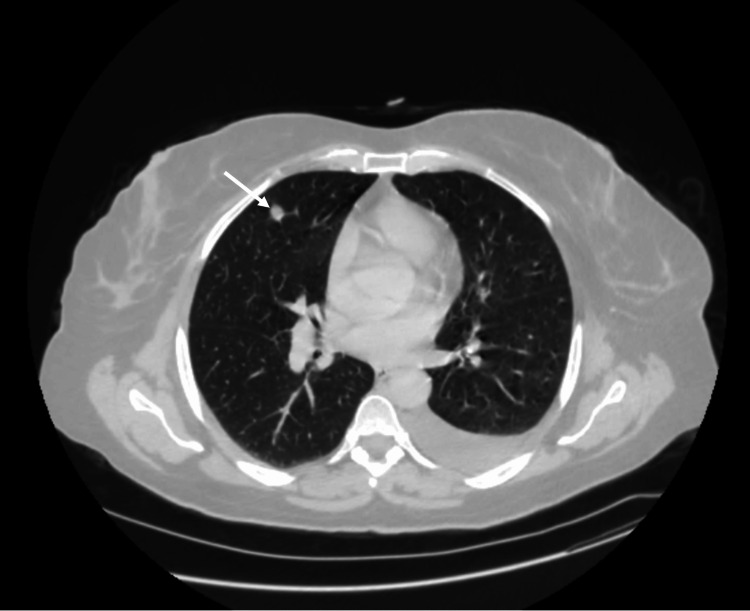
CT scan image showing a pulmonary metastasis

**Figure 3:**
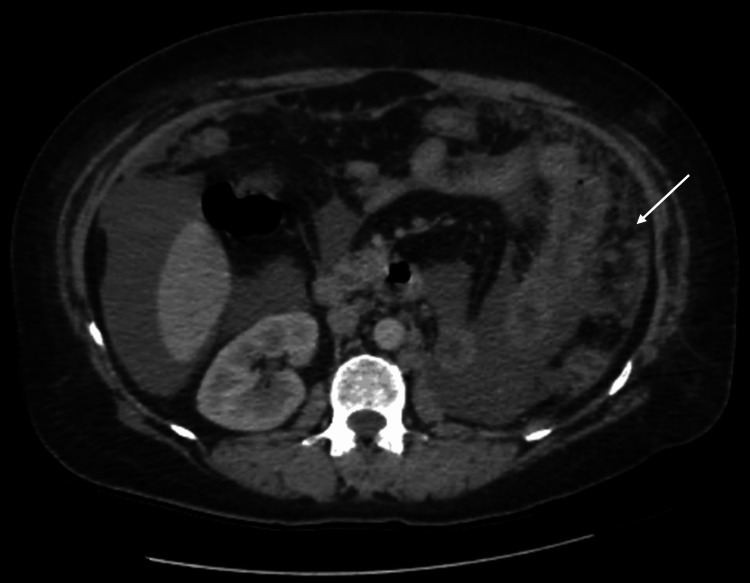
Axial CT scan showing a stranding pattern of the peritoneal fat consistent with peritoneal carcinomatosis

**Figure 4:**
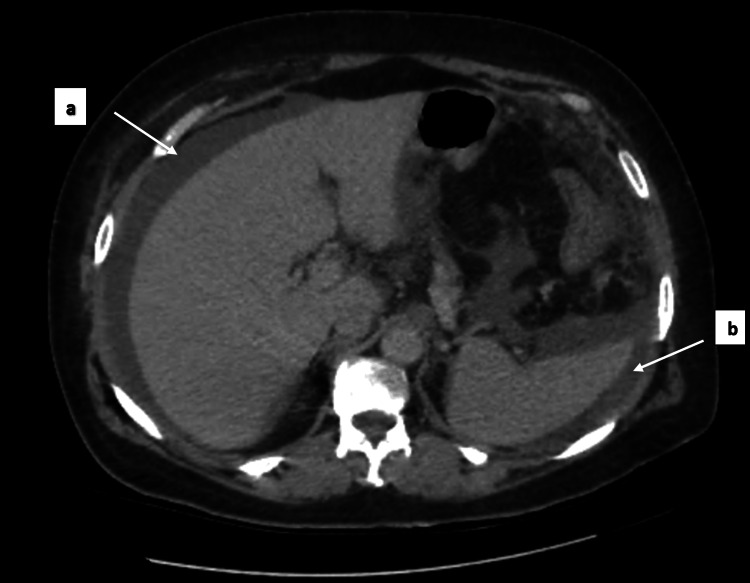
Axial CT scan showing perihepatic (a) and perisplenic (b) fluid collections

**Figure 5:**
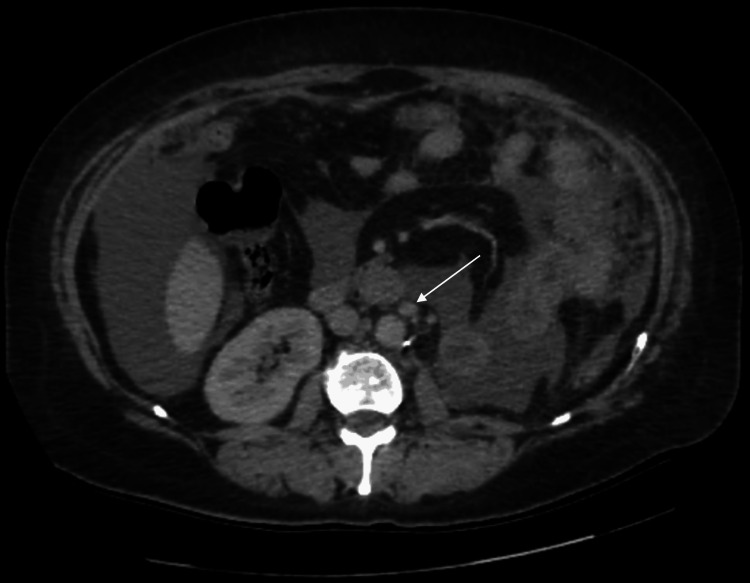
Axial CT scan showing preaortic lymph nodes

**Figure 6:**
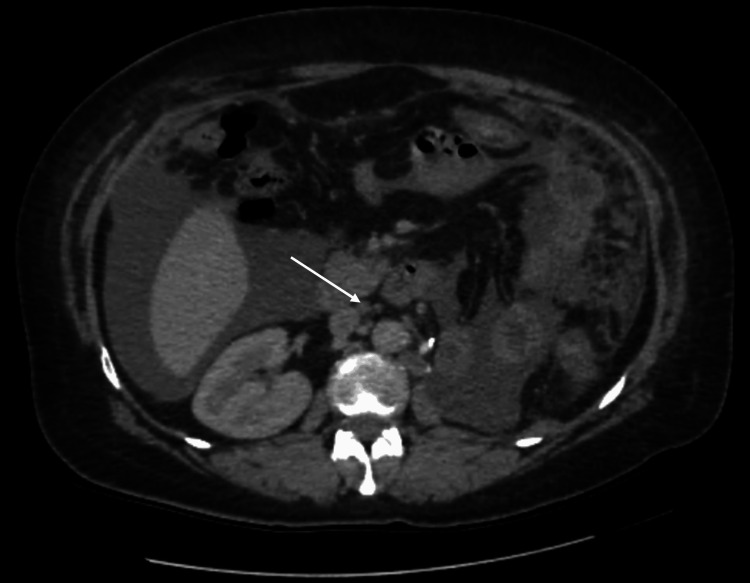
Axial CT scan showing latero-aortic lymph nodes

A contrast-enhanced CT urogram revealed a large tumor mass in the mid-region of the left kidney (Figure 1), measuring 104 × 96 × 111 mm, associated with left latero-aortic lymphadenopathy. A thoraco-abdominopelvic CT scan performed for staging revealed multiple bilateral pulmonary nodules and micronodules (Figure 2), diffuse micronodular and flame-like infiltration of the peritoneal fat (Figure 3), and moderate ascites located perihepatically, perisplenically, in both flanks, iliac fossae, and the pelvis (Figure 4). Additionally, several preaortic (Figure 5) and left latero-aortic lymphadenopathies (Figure 6) were observed, the largest measuring 15 mm in the short axis.

**Figure 7:**
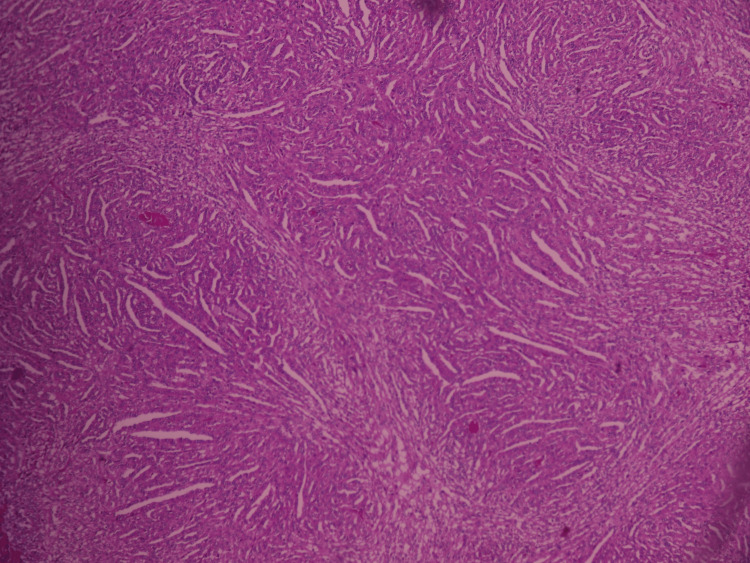
Low magnification (×100) of carcinomatous proliferation Carcinomatous proliferation is composed of irregularly arranged and elongated tubular structures, with occasional glandular and solid areas. The tumor cells are cuboidal, resembling upholstery tack cells, and exhibit a high nuclear-to-cytoplasmic ratio. A peritubular and periglandular inflammatory infiltrate is noted at the periphery.

**Figure 8:**
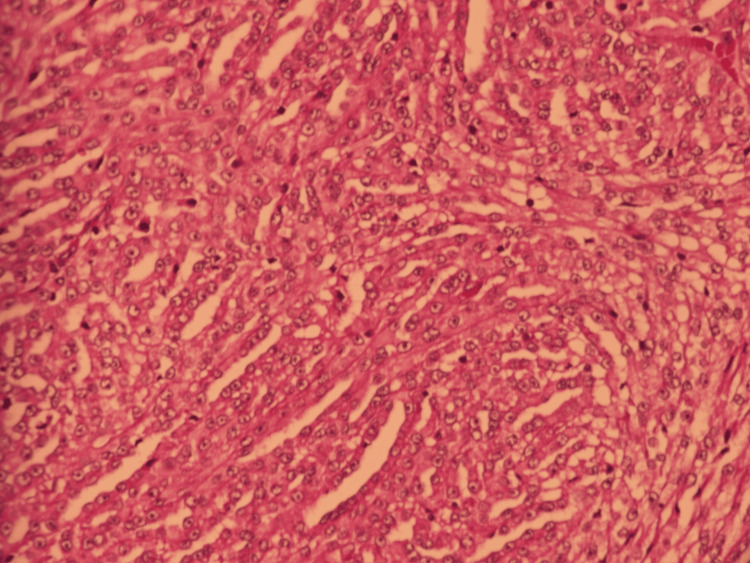
High magnification (×400) of carcinomatous proliferation Carcinomatous proliferation is composed of irregularly arranged and elongated tubular structures, with occasional glandular and solid areas. The tumor cells are cuboidal, resembling upholstery tack cells, and exhibit a high nuclear-to-cytoplasmic ratio. A peritubular and periglandular inflammatory infiltrate is noted at the periphery.

**Figure 9:**
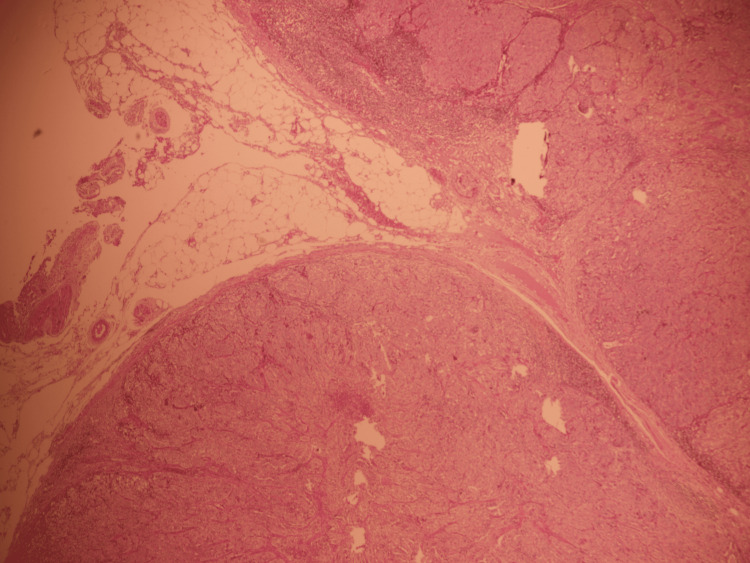
Low magnification of anglionic infiltration

**Figure 10:**
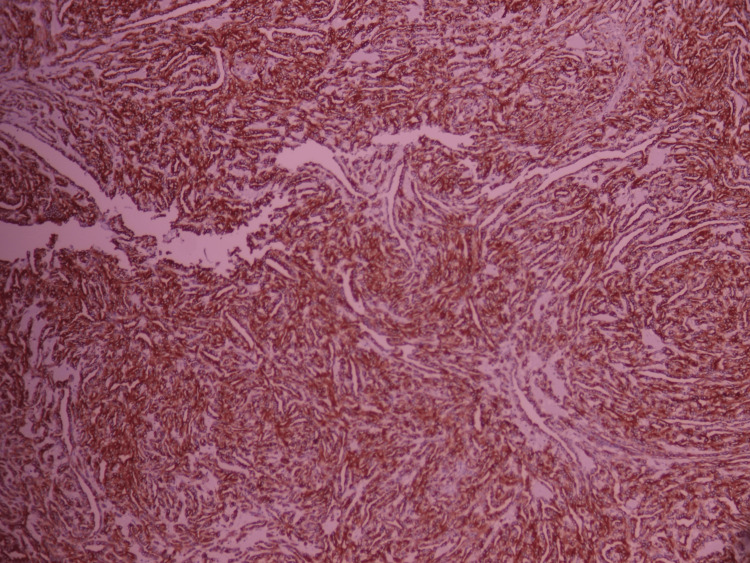
Membranous positivity for anti-cytokeratin 7, with diffuse expression

**Figure 11:**
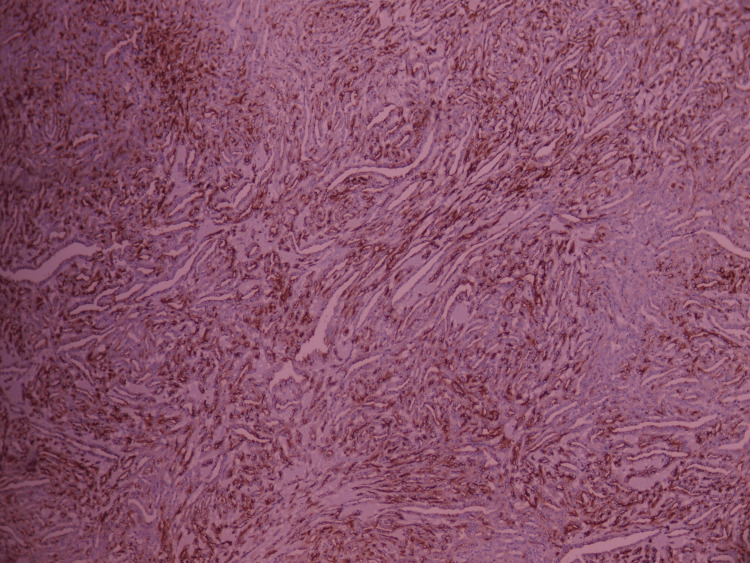
Membranous positivity for anti-epithelial membrane antigen, with diffuse staining

Histopathological analysis of the nephrectomy specimen showed a carcinomatous proliferation composed of elongated, irregularly arranged tubules, with occasional large tubular structures. The tumor cells were cuboidal, with a “hobnail” appearance and a high nuclear-to-cytoplasmic ratio. Periglandular and peritubular infiltration was noted at the periphery, along with multiple vascular and lymphatic emboli located at the renal hilum and capsule (Figure 7, Figure 8). Nerve sheath infiltration was also observed. The perirenal fat was free of tumor invasion; however, the renal vein was infiltrated. All lymph nodes from the lymphadenectomy specimen were tumor-positive (Figure 9), with capsular rupture observed in one node. The diaphragmatic nodule was also found to be carcinomatous. Immunohistochemical analysis showed positive staining for cytokeratin 7 (Figure 10) and epithelial membrane antigen (Figure 11), while HER2, PAX8, vimentin, and GATA2 were negative. These findings were consistent with a diagnosis of Bellini duct carcinoma.

Chemotherapy was initiated based on a gemcitabine-cisplatin regimen (gemcitabine 1000 mg/m² on days 1 and 8; cisplatin 25 mg/m² on days 1, 2, and 3; cycles repeated every 21 days), which was well tolerated. The patient responded favorably to treatment, with clinical and radiological stability after three cycles.

## Discussion

CDC of Bellini is a rare renal tumor, accounting for approximately 1% [1] of epithelial renal malignancies. It typically arises in middle-aged patients, with an average age of onset around 55 years, consistent with the age of the patient in our case. A male predominance has been reported, with a male-to-female ratio of approximately 2:1 [2]. Chao et al. [3] observed that nearly half of patients diagnosed with CDC had a family history of cancer; however, this was not the case for our patient.

CDC most commonly presents symptomatically [3], with clinical manifestations dominated by macroscopic hematuria in 50-66.7% of cases, lumbar pain in 40%, and occasionally a palpable mass in the lumbar region or flank [1]. Radiologically, Bellini duct carcinoma lacks specific imaging characteristics [4]. Tumors are often poorly enhanced after contrast injection, indicating relative hypoperfusion. Furthermore, there is a frequent association with renal vein or inferior vena cava thrombus [5].

The diagnosis of CDC is based on histopathological examination. Macroscopically, the tumor is usually centered in the renal medulla. It appears large, firm, yellow or grayish on the cut section, poorly circumscribed, and often associated with satellite nodules and hemorrhagic changes [4]. Microscopically, it presents as a glandular tumor composed of irregularly contoured tubules with medullary distribution. The tumor cells are typically large, with eosinophilic cytoplasm, prominent nucleoli, and high nuclear grade [4]. According to Fondimare et al. [4], a combination of gross morphology with tubular, microcystic, and papillary architecture, along with cytological features, strongly supports the diagnosis. Srigley and Eble [6] proposed a diagnostic framework including five major and four minor histological criteria for confirming CDC.

Radical nephrectomy remains a component of the therapeutic approach, including in metastatic patients, although its benefit is limited due to the high perioperative and immediate postoperative mortality risk. Therefore, in the presence of a large, symptomatic, and infiltrative renal mass identified on CT imaging, a preoperative tumor biopsy is recommended to guide management [5].

Chemotherapy remains the most commonly used first-line treatment, typically based on a platinum salt (cisplatin or carboplatin) combined with gemcitabine (the CG regimen). This approach is supported by the results of a prospective phase II trial conducted by the French Genitourinary Tumor Group (GETUG) [7]. Several teams, considering the histological similarities between CDC and urothelial carcinoma, have tested the MVAC regimen (methotrexate, vinblastine, doxorubicin, and cisplatin) without success [5]. A single case report in the literature described a temporary response to the combination of Adriamycin and gemcitabine [8].

The prognosis of Bellini duct carcinoma is extremely poor [5]. Approximately 50% of cases present with metastatic disease at the time of diagnosis. In metastatic patients, the average overall survival is eight months, with a median survival of six months [5].

## Conclusions

CDC of Bellini is a rare malignant renal tumor characterized by poor prognosis, primarily due to frequent diagnosis at a metastatic stage. The diagnosis relies mainly on histological analysis, supplemented by immunohistochemical studies. Although radical nephrectomy may be indicated in select cases, it has not demonstrated a significant impact on prognosis in metastatic settings. Consequently, first-line treatment currently relies on a combined approach, primarily involving gemcitabine-cisplatin-based chemotherapy within multicenter clinical protocols, aiming to improve the management and outcomes of this aggressive tumor.
